# What is your diagnosis?

**Published:** 2011-09-25

**Authors:** L. Aghaghazvini, P. Karami, O. Yeganeh, Sh. Aghaghazvini

**Affiliations:** 1 Assistant Professor, Department of Radiology, Shariati Hospital, Tehran University of Medical Sciences, Tehran, Iran; 2 Resident of Radiology, Tehran University of Medical Sciences, Tehran, Iran; 3 Advanced Diagnostic and Interventional Radiology Research Center (ADIR), Imam Khomeini Hospital, Tehran University of Medical Sciences, Tehran, Iran

A 59-year-old woman was referred with nausea, vomiting and generalized abdominal pain after colonoscopy. She is a known case of amyloidosis for 10 years that resulted in renal failure. (Figs. 1. A-F)

**Fig. 1:**
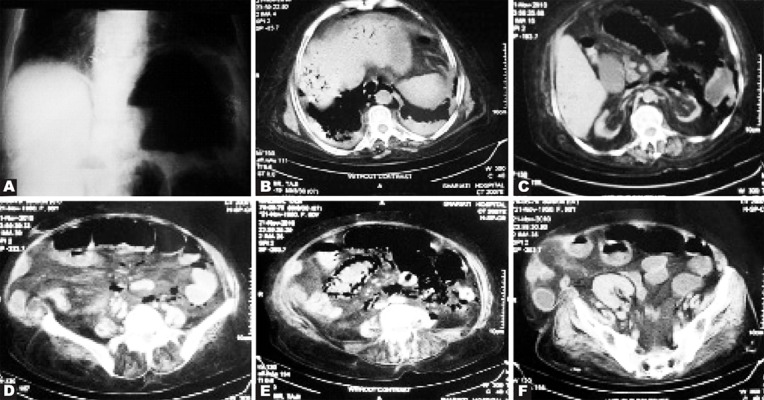
A 59-year-old woman with nausea, vomiting and generalized abdominal pain after colonoscopy. A. Plain abdominal graphy shows dilation of bowel loops and air in the subdiaphragmatic and retroperitoneal area. B-F. Contrast enhanced abdominopelvic CT scan shows free air in the retroperitoneal, intraperitoneal and posterior mediastinal spaces with pneumatosis intestinalis and gas bubble in the portal vein branches. Note the transplant kidney and atrophic native kidneys and right abdominal wall incisional hernia. Bilateral pleural effusion and ascites is evident too.

## Diagnosis: Post Colonoscopic Bowel Perforation in Underlying Secondary Amyloidosis

Amyloidosis is a rare disease in which extracellular fibrillar proteins deposit in tissues and organs interfering with the normal functioning of the affected organ.

This disease is defined based on the biochemical nature of fibril proteins. The collection of abnormal proteins may be localized in one organ or may be systemic and involve many organs of the body. Secondary amyloidosis refers to a group of diseases in which a composition of the acute phase reactant serum amyloid A protein accumulates in tissues and organs in association with chronic inflammatory disease.[1] Although many inflammatory conditions may lead to secondary amyloidosis, it is a rare complication in psoriasis and unfortunately associated with a poor outcome.[2]

The gastrointestinal tract (especially the small bowel) is usually involved by systemic amyloidosis and deposition of amyloid in the intestinal wall leads to a variety of signs and symptoms such as abdominal pain, dysmotility, pseudoobstruction, GI bleeding and malabsorbtion. However, small bowel infarction and perforation is also a rare complication of intestinal involvement.[3] It seems that deposition of amyloid in the vessels of the small intestinal wall leads to ischemia, necrosis and perforation of the bowel wall.[1][3] This rare complication could be fatal.

Some authors believe that because of the poor prognosis of spontaneous intestinal perforation in amyloidosis and the fact that the same results are achieved in conservative and operative management, surgical procedure should be limited to acute abdomen.[4][5]

Our case was a 59-year-old woman with a history of secondary amyloidosis on an underlying psoriasis disease for 10 years who presented with severe generalized abdominal pain, nausea and vomiting 12 hours following colonoscopy. This procedure was performed as the standard procedure for determining the cause of anemia in this patient after positive fecal occult blood exam. She had undergone living donor renal transplantation because of end stage renal disease 2 years ago.

On physical examination, she had unstable vital signs (systolic blood pressure, 70 mmHg; pulse rate, 90 b/ min) and fever. Abdominal examination revealed generalized abdominal tenderness and there was an abdominal wall hernia near the transplant kidney scar.

Laboratory tests showed normal limit levels except for leukocytosis (WBC, 17400; PMN, 70%).

Ultrasound showed a small amount of free fluid in the peritoneal cavity with mildly dilated bowel loops. There was a fascial defect near the recent surgical scar and fluid-filled small-bowel loop and a small amount of free fluid was seen in this hernia.

Plain abdominal graphy showed dilatation of bowel loops with air in the subdiaphragmatic and retroperitoneal spaces.

Contrast enhanced abdominopelvic CT scan revealed dilatation of the bowel loops (mainly the small bowel) with pneumatosis intestinalis and free air in the retroperitoneal, intraperitoneal and posterior mediastinal spaces with hepatic portal venous gas. In addition, atrophic native kidneys and transplant kidney in the right pelvic cavity and adjacent incisional hernia containing bowel loops were evident. Bilateral pleural effusion and ascites were detected.

According to the above mentioned findings, spontaneous perforation due to bowel necrosis secondary to amyloidosis or perforation following colonoscopy were our differential diagnoses.

In this case, regarding the history of recent colonoscopy, it was doubtful that perforation was due to bowel wall involvement by amyloidosis or occurred after the colonoscopy procedure; however, the second diagnosis was more probable.

As a result of this finding and the unstable condition of the patient, conservative treatment was started initially before surgery, but the patient did not tolerate and unfortunately expired after a few hours.

This case was interesting because of not only the rarity of secondary amylodosis due to psoriasis, but also severe probable involvement of the bowel loops by the disease and perforation following colonoscopy (not primary).
